# New Approaches in Characterization of Lesions Dissemination in DLBCL Patients on Baseline PET/CT

**DOI:** 10.3390/cancers13163998

**Published:** 2021-08-08

**Authors:** Anne-Ségolène Cottereau, Michel Meignan, Christophe Nioche, Jérôme Clerc, Loic Chartier, Laetitia Vercellino, Olivier Casasnovas, Catherine Thieblemont, Irène Buvat

**Affiliations:** 1 Department of Nuclear Medicine, Cochin Hospital, AP-HP, University of Paris, 75014 Paris, France; jerome.clerc@aphp.fr; 2 LITO Laboratory, U1288, Institut Curie, Université PSL, Inserm, Université Paris Saclay, 91400 Orsay, France; christophe.nioche@curie.fr (C.N.); irene.buvat@curie.fr (I.B.); 3 LYSA Imaging, Henri Mondor University Hospital, AP-HP, University Paris East, 94000 Créteil, France; michel.meignan-ext@aphp.fr; 4 The Lymphoma Academic Research Organisation, Statistic, Centre Hospitalier Lyon Sud, 69000 Pierre-Benite, France; loic.chartier@lysarc.org; 5 Department of Nuclear Medicine, Saint-Louis Hospital, AP-HP, 75010 Paris, France; laetitia.vercellino@aphp.fr; 6 Department of Hematology, University Hospital of Dijon, 21231 Dijon, France; olivier.casasnovas@chu-dijon.fr; 7 Department of Hematology, Saint-Louis Hospital, AP-HP, Hemato-Oncology, DMU DHI, 1 Av. Claude Vellefaux, 75010 Paris, France; catherine.thieblemont@aphp.fr; 8 Research Unit NF-kappaB, Différenciation et Cancer, Université de Paris, 12 Rue de l’École de Médecine, 75006 Paris, France

**Keywords:** dissemination metrics, FDG-PET/CT, DLBCL, SDMax

## Abstract

**Simple Summary:**

Recently, a new PET parameter expressing lymphoma dissemination has been proposed to identify high-risk DLBCL patients: the distance between the two furthest lesions, standardized by body surface area (SDmax). This study aimed to determine the best way to measure the distance between lesions, by comparing different methods of distance measurements. We obtained similar results in terms of prediction of outcome between the different methods further validating the relevance of the dissemination features. We highlighted the possibility to calculate it directly from lymphoma voxels instead of lesion centroids, and thus applied it to a metabolic tumor volume (MTV) determined by deep learning algorithms. This could allow the use in clinical practice of this parameter, characterizing tumor spread, in combination with the tumor burden, for patient risk stratification.

**Abstract:**

Dissemination, expressed recently by the largest Euclidian distance between lymphoma sites (SDmax), appeared a promising risk factor in DLBCL patients. We investigated alternative distance metrics to characterize the robustness of the dissemination information. In 290 patients from the REMARC trial (NCT01122472), the Euclidean (Euc), Manhattan (Man), and Tchebychev (Tch) distances between the furthest lesions, firstly based on the centroid of each lesion and then directly from the two most distant tumor voxels and the Travelling Salesman Problem distance (TSP) were calculated. For PFS, the areas under the ROC curves were between 0.63 and 0.64, and between 0.62 and 0.65 for OS. Patients with high SDmax whatever the method of calculation or high SD_TSP had a significantly poorer outcome than patients with low SDmax or SD_TSP (*p* < 0.001 for both PFS and OS), with significance maintained in Ann Arbor advanced-stage patients. In multivariate analysis with total metabolic tumor volume and ECOG, each distance feature had an independent prognostic value for PFS. For OS, only SDmax_Tch, SDmax_Euc _Vox, and SDmax_Man _Vox reached significance. The spread of DLBCL lesions measured by the largest distance between lymphoma sites is a strong independent prognostic factor and could be measured directly from tumor voxels, allowing its development in the area of the deep learning segmentation methods.

## 1. Introduction

Tumor dissemination is a well-known prognostic factor in solid tumors but also in lymphoma disease, where it is expressed by Ann Arbor classification first described for Hodgkin lymphoma [1] but rapidly extended to non-Hodgkin lymphoma [2], especially Diffuse Large B Cell Lymphoma (DLBCL). DLBCL is an aggressive lymphoma characterized by significant heterogeneity of clinicopathologic and molecular genetic features. Early-stage DLBCL, categorized as Stage I or Stage II, accounts for about 25% of all patients with DLBCL and is known as “limited stage lymphoma”. Advanced stage DLBCL, namely stages III and IV, accounts for 75% of DLBCL patients with 5-year survival rates with R-CHOP treatment of about 50–55% [3]. Despite great efforts to identify better treatment options for DLBCL to control the disease and prevent relapse, 10 to 15% of patients have primary refractory disease within 3 months after treatment initiation and another 20 to 35% relapse [4] even if they are in complete response after first-line immunochemotherapy [5]. New biomarkers are therefore needed for stratifying patients according to risk factors and for providing insights into the use of more targeted and individualized therapeutics. Baseline quantitative PET metrics could be used to better characterized lymphoma disease. The total metabolic tumor volume (TMTV) reflecting the whole tumor burden has been widely investigated and showed high prognostic value [6,7,8,9]. More recently, the dissemination of this TMTV has been expressed by calculating the distance between the different lesions in the whole body [10]. The centroid of each lesion, i.e., each volume of interest (VOI) was considered as the lesion location and Euclidean distance was used. The largest distance between lymphoma sites, called Dmax, was found to have a high prognostic impact on DLBCL outcome [10,11]. Until now TMTV was obtained by summing each individual lesion delineated with a semi-automated image analysis software, allowing the location of each individual lesion and subsequent calculation of distance between lesions. However, the development of fully automatic segmentation algorithms based on convolutional neural networks is growing [12], providing directly the TMTV value without separating the individual lesions and thus preventing the calculation of Dmax based on lesion centroids. The aim of this study was first to explore if other distance features, such as the shortest distance that connects all patient lesions, may improve the prediction of prognosis and secondly if the maximal distance between the two furthest lesions could be extracted directly from the two most distant tumor voxels, in a large prospective cohort of DLBCL patients. 

## 2. Materials and Methods

### 2.1. Patients

Patients from an ancillary study of the REMARC trial (NCT01122472) [13] who had a baseline PET/CT available for retrospective review were analyzed. A total of 290 patients were selected, with Body Surface Area (BSA) data and with at least 2 detectable lesions allowing distance measurement [11]. The REMARC study design and details have been reported elsewhere [5]. Briefly, DLBCL patients who were 60–80 years old, and had a complete response or partial response as defined by Cheson 2007 criteria [14] after 6 or 8 cycles of standard R-CHOP were included in that trial and randomized 1:1 to Lenalidomide maintenance or placebo for 21 of every 28-day cycle for 2 years. The cell of origin was also determined in a subset of patients by molecular testing from formalin-fixed paraffin-embedded tissue using NanoString gene expression profiling (GEP) technology. The institutional review board approval and the informed consent of the REMARC trial included all the ancillary studies.

### 2.2. PET/CT Scanning

All the centers followed EANM guidelines for patient preparation and PET/CT acquisition. The delay between 18F-FDG injection and acquisition time was 71.7 ± 14.1 min (mean ± std). Whole-body PETs were acquired using different scanner models from different vendors, as summarized previously [15].

Baseline 18F-FDG PET/CT images were collected in an anonymized Digital Imaging and Communications in Medicine (DICOM) format. Patients with incomplete axial slices or irregular slice intervals were excluded.

### 2.3. PET/CT Analysis

**Figure 1:**
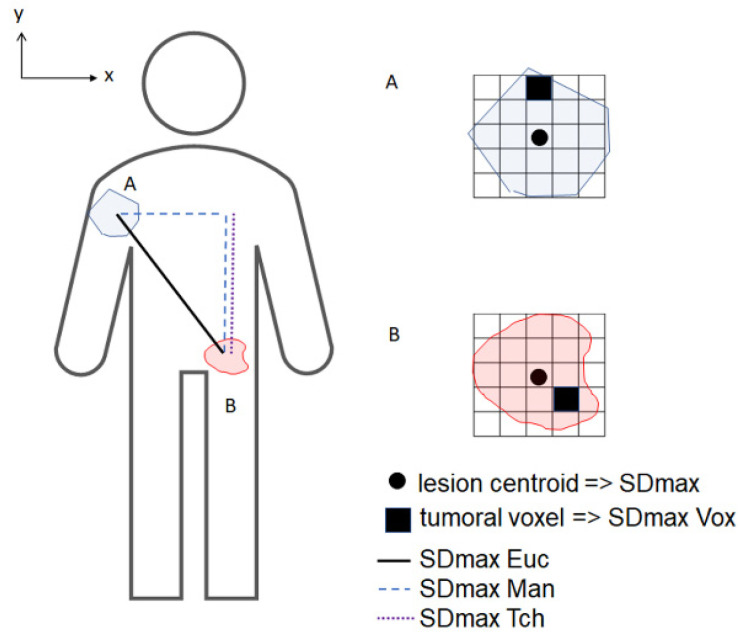
Schematic PET coronal image illustrating the 3 distance definitions in 2D between the 2 furthest lesions A (x_A_,y_A_) and B (x_B_,y_B_).

Lymphoma regional volumes were automatically identified by the software Beth Israel Fiji (http://petctviewer.org, accessed on 7 August 2021) and then checked visually to confirm the inclusion of only pathological lesions [16] by two nuclear medicine physicians (ASC, LV) blinded to patient outcome. Metabolic tumor volumes were defined using a 41% SUVmax threshold. Bone marrow involvement was included in the volume measurement only if there was focal uptake as previously described [17]. Different distance features between all pairs of lesions (including both nodal and extranodal lesions) were extracted from LIFEx software [18]. Firstly, different distances between the center of mass (centroid) of each lesion were calculated. Three different types of maximal distance (Figure 1) were tested: (1) Euclidean distance: $\mathrm{AB}=\sqrt{{\left(x_{B}-x_{A}\right)}^{2}+{\left(y_{B}-y_{A}\right)}^{2}+{\left(z_{B}-z_{A}\right)}^{2}}$ [10] (2) Manhattan distance: $\mathrm{AB}=|x_{B}-x_{A}\left|+\right|y_{B}-y_{A}\left|+\right|z_{B}-z_{A}|$ (3) Tchebychev distance $\mathrm{AB}=\max\;(|x_{B}-x_{A}\left|,\;\right|y_{B}-y_{A}\left|,\;\right|z_{B}-z_{A}\left|\right)$. In addition, the shortest distance that connects all patient lesions was calculated as a solution to the Travelling Salesman Problem (TSP) using a heuristic method (Google OR-tool, https://developers.google.com/optimization/routing/tsp, accessed on 7 August 2021), corresponding to the shortest possible loop that connects all lesion centroids. All distances were normalized by the BSA, given by the formula $\sqrt{\left(\mathrm{weight}\;\times \;\mathrm{height}\right)/3600}$, yielding standardized distances (SD). The resulting maximum distances (SDmax) were noted SDmax_Euc (Euclidian distance), SDmax_Man (Manhattan distance), SDmax_Tch (Tchebychev distance), and SD_TSP (TSP distance). Secondly, we have evaluated the maximal distances calculated directly from the tumor voxels (volume element of a three-dimensional space), ie the distance between the 2 most distant voxels belonging to the total MTV, regardless of the lesion each voxel belongs to (noted SDmax_Euc_vox, SDmax_Man_vox, and SDmax_Tch_vox).

### 2.4. Statistical Analysis

PFS was measured from the date of randomization to the date of death from any cause, disease relapse or progression, or the date of the last contact. OS was calculated from the date of randomization until the date of death from any cause or the date of the last contact. For each method, receiver-operating-characteristic (ROC) curves were calculated. The optimal cut-off for PFS and OS was determined by ROC and X-tile analyses. The performances of each method to predict PFS and OS were pairwise compared by comparing the areas under the ROC curves (AUC, DeLong test). Survival functions for both PFS and OS were calculated with Kaplan–Meier (KM) estimates for each method using their optimal PFS SDmax cut-off. KM curves were compared using the log-rank test and Cox proportional hazard models. Multivariate analyses were performed using Cox proportional hazard models including each SDmax with MTV and ECOG, as these two factors have been previously published as the best independent prognosticators [19]. The Chi-squared test was used to test the relationship between Ann Arbor classification and PFS/OS events in low vs high SDmax groups. Test results were interpreted as significant if the 2-sided *p*-value was less than 0.05. Statistical analyses were conducted using MedCalc software (MedCalc Software, Ostend, Belgium), SAS 9.3, and X-tile 3.6.1 software (Yale University, New Haven, CT, USA).

## 3. Results

### 3.1. Patient Characteristics

The 290 patients baseline characteristics were previously reported [11]. Briefly, half of them (52%, *n* = 150) belonged to the Lenalidomide arm, 91% (*n =* 264) had an advanced stage, 71% (*n* = 205) had an IPI ≥ 3, 16% (*n =* 47) ECOG ≥ 2, 60% elevated LDH upper than normal (*n* = 175), 51% (*n =* 147) extranodal sites ≥ 2, and 55% (*n =* 158) had an MTV ≥ 220 cm^3^. Among this cohort, 190 patients had molecular analysis using GEP: 96 were classified as GCB (50.5%), 71 ABC (37.4%) and 23 unclassified (12.1%).

### 3.2. PET Characteristics

The descriptive statistics obtained for SDmax and SD_TSP calculated using the different distance definitions are given in Table 1.

**Figure 2:**
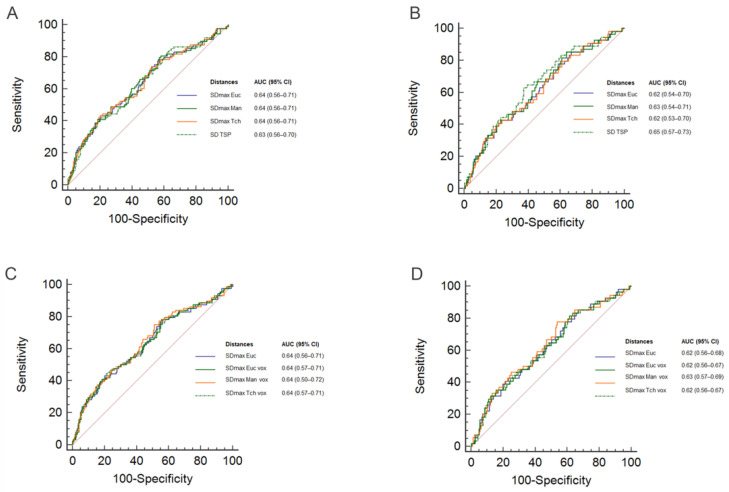
Receiver Operating Curves (ROC) for SDmax_Euc, SDmax_Man, SDmax_Tch, SD_TSP for PFS (**A**), and OS (**B**), for SDmax_Euc, SDmax_Euc_Vox, SDmax_Man_Vox, SDmax_Tch_Vox for PFS (**C**) and OS (**D**). The tables show the area under the curve (AUC) with 95% confidence intervals (95% CI).

For both PFS and OS, the areas under the Receiver Operating Curves (ROC AUC) obtained using SDmax_Man and SDmax_Tch were not significantly different from the area under the curve corresponding to SDmax_Euc (Figure 2A,B; pairwise comparison: *p* > 0.7 for PFS, *p* > 0.1 for OS). Similarly, the AUC obtained using SDmax based on the voxels (SDmax_Euc_vox, SDmax_Man_vox, and SDmax_Tch_vox) were not significantly different from the AUC corresponding to SDmax_Euc for both PFS and OS (Figure 2C,D; pairwise comparison *p* > 0.7 for PFS, *p >* 0.1 for OS). Cohen’s Kappa coefficient (with SDmax_Euc as reference) ranged from 0.82 to 0.92 (Table 2).

**Figure 3:**
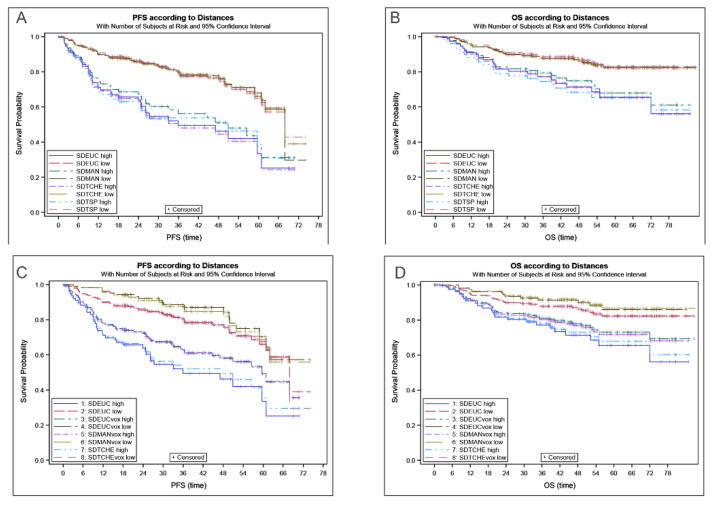
Kaplan–Meier curves for SDmax_Euc, SDmax_Man, SDmax_Tch, SD_TSP for PFS (**A**) and OS (**B**), for SDmax_Euc, SDmax_Euc_Vox, SDmax_Man_Vox, SDmax_Tch_Vox for PFS (**C**) and OS (**D**).

High SDmax was significantly associated with poorer outcomes whatever the method of calculation (Table 2). Patients in the low SDmax group had significantly longer PFS and OS than patients in the high SDmax group using the optimal PFS/OS derived cut-off, with similar separation between high and low SDmax groups for all methods (Figure 3). Results obtained with SDmax calculated directly from the voxels showed a slight improvement of outcome prediction (Table 2).

Regarding the SD_TSP, measuring the length of a path through all lesions, the ROC curve was similar to the SDmax curves and did not show superiority for outcome prediction (Table 2, Figure 3A,B).

### 3.3. Comparison with Other Prognosticators

In multivariable analysis with MTV and ECOG (Table 3), each distance feature had an independent prognostic value for PFS. For OS, only SDmax_Tch, SDmax_Euc _Vox, and SDmax_Man _Vox reached significance.

In a sub-analysis of Ann Arbor advanced III–IV stage patients (*n =* 264), each distance remained a significant prognosticator for both PFS (*p* < 0.0001 for SDmax_Euc, *p* = 0.007 for SDmax_Man, *p* < 0.0001 for SDmax_Tch, *p* = 0.0015 for SD_TSP) and OS (*p* = 0.0059, *p* = 0.0178, *p* = 0.0071 and *p* = 0.0010 respectively).

In the low SDmax_Euc group, we observed no significant differences in the number of PFS (*p* = 0.24) or OS (*p* = 0.55) events regardless of Ann Arbor stage: 16% of PFS events stage II patients, 26% in stage III patients and 24% in stage IV patients; 8%, 12% and 17% of OS events respectively. 

In high SDmax_Euc, PFS events were observed in 31% of stage III patients and 49% of stage IV patients (*p* = 0.35), OS events were observed in 8% and 32% respectively (*p* = 0.15). No patients were stage I–II.

## 4. Discussion

We have shown in a series of 290 DLBCL patients included in a prospective trial that the spread of the lymphoma lesions measured on baseline PET by the distance between the lesions that were the furthest apart was a robust prognostic factor whatever the methods used for distance measurement.

First, we have compared different distance definitions to calculate the largest normalized distance (SDmax) between the centroid of lymphoma sites. The distribution and area under the ROC curves for the three distance definitions were similar, suggesting the methods to be close in accuracy for the prediction of outcome. Kaplan–Meier analyses showed that the patients with low SDmax have a significantly longer 4y-PFS compared to the patients with high SDmax, regardless of the method. This result further validated the relevance of the dissemination features [10] whatever the metrics used to assess it.

We also tested the prognostic value of another distance feature, the shortest route between all the patient lesions, called “the TSP distance”, as another way to express the spread of the lesions. Unlike SDmax, this parameter took into account the number of lesions, as a partial reflection of tumor burden. However, we did not find any advantage in using this feature compared to the largest distance between lymphoma sites for prognostication. Especially when combined with MTV as the most accurate tumor burden surrogate, SDmax, reflecting only the spatial distribution, appeared more complementary.

Since the maximal distance between two lymphoma lesions is prognostic using the center of gravity of each lesion, we decided to test if it remains prognostic when considering the two most distant points, i.e. the two most distant voxels, belonging to the total MTV. We showed that the prognostic value of SDmax_Vox persisted and was even slightly increased in comparison with SDmax calculated from the centroid of the furthest lesions. Indeed, SDmax_Vox slightly majored the maximal distance. This is important as some tumor segmentation methods involving deep learning only highlight tumor voxels without assigning voxels to a specific lesion [12,20]. In addition, this could make theoretically possible a rough evaluation of the distance on a Maximum Intensity Projection image displayed in 2D. Our results suggest that the proposed dissemination metrics remain an effective predictor of PFS and OS even when the different tumor foci are not identified. 

The TSP distance cannot be calculated based on the voxels belonging to lesions and needs the identification of separate lesion foci and associated lesion centroids for computational tractability. Yet, the TSP distance did not appear to be superior to the other distances calculated in our cohort so not being able to calculate it based on tumor voxels instead of tumor foci does not appear to be a limitation.

Ann Arbor classification till now is the standard for lymphoma staging, with minor modifications introduced by the Lugano classification [21,22]. Distance measurements whatever the way of expression used to calculate them perform better than the Ann Arbor score for prognostic evaluation and could be proposed as an alternative given it is easy to calculate. It remained significant in advanced-stage patients. No significant difference of outcome was observed between the different stages in the group “low distance” on one hand, and in the “high distance” group on the other hand. The dissemination expressed in our study does not explain the pathophysiology of the spread of the lesions observed in DLBCL. We have tested the predictive power of these dissemination biomarkers in DLBCL only and investigating their predictive values for other types of lymphomas is needed to determine whether these distances might reflect specific pathophysiological patterns. It would be important to compare these dissemination parameters to another index of lymphoma spread such as circulating tumor DNA level in the blood which proved to be a prognostic factor in DLBCL patients [23] and to investigate if one or the other is better linked to the different molecular subtypes recently described in DLBCL [24,25]. Large studies are needed to determine more accurately the impact of SDmax in each DLBCL subtype.

The combination of MTV and ECOG performance status has been proposed to identify high-risk DLBCL patients at baseline. Recently, this combination has been validated in a series of 2306 DLBCL patients [26], including 1796 patients treated in clinical trials in Europe and the United States and 481 patients treated in real-life situations across multiple centers in Europe. It improves risk stratification for patients with DLBCL treated in frontline by standard treatment or intensified immuno-chemotherapy. In the current study, multivariable analysis with MTV and ECOG showed that SDmax_Euc_Vox had independent prognostic value for both PFS and OS. We suggest that SDmax could complement the MTV ECOG score since they are two different ways to characterize the lymphomatous lesions in DLBCL. Consistent with our findings, recently, Eertink et al. [27] have shown in 317 DLBCL patients included in the HOVON84 trial [28] that combining radiomics features, such as MTV and the Dmax_bulk_, with clinical predictors, increased the positive predictive value with a more accurate selection of high-risk patients compared to the IPI model. Similarly, in 133 patients included in the SAKK38/07 trial (NCT00544219) Dmax was one of the four parameters included in the proposed prognostic score, among more than > 100 extracted radiomic features [29]. In addition, the prognostic value of dissemination, expressed by Dmax, has been also demonstrated recently in Hodgkin lymphoma [30]. The strength of our study is to define a simple radiomic feature and its different types of measurement, which could be applied successfully in other subtypes of lymphoma. It would be also interesting to explore its role in solid metastatic tumors. Obviously and it is the limitation of the work, the cut-off found in this cohort cannot at this time be considered as a gold standard and should be validated in other larger studies.

## 5. Conclusions

To conclude, we obtained similar results between the different distances used to characterize tumor spread in terms of prediction of outcome further validating the relevance of the dissemination features measured on baseline PET/CT for DLBCL patients. We have shown that a distance metric including the number of sites did not improve the prognostic value. We underlined the possibility to calculate the maximal distance directly from lymphoma voxels instead of lesion centroids, which could be applied on an MTV determined by machine learning or deep learning algorithms.

## Figures and Tables

**Table 1:** Descriptive statistics for the 7 dissemination features.

Standardized Distances (m^−1^)	Mean	Median	SD	Q1–Q3
SDmax_Euc	0.230	0.232	0.12	0.13–0.33
SDmax_Man	0.296	0.293	0.15	0.17–0.41
SDmax_Tch	0.223	0.225	0.12	0.12–0.32
SD_TSP	0.765	0.657	0.56	0.34–1.03
SDmax_Euc_Vox	0.248	0.249	0.12	0.14–0.34
SDmax_Man_Vox	0.326	0.317	0.15	0.21–0.43
SDmax_Tch_Vox	0.240	0.245	0.12	0.13–0.34

**Table 2:** Log-rank scores from a comparison of high/low standardized maximal distances (SDmax) for each method (Euclidian, Manhattan, Tchebychev, Travelling Salesman Problem distance, and Euclidian, Manhattan, Tchebychev voxels based) with the number of events, 4-year PFS (4y-PFS) and 4-year OS (4y-OS). Log-rank scores revealed significant differences in PFS and OS between high/low SDmax with all methods.

Method		SDmax _Euc	SDmax _Man	SDmax _Tch	SDTSP	SDmax _Euc_Vox	SDmax _Man_Vox	SDmax _Tch_Vox
Kappa	SDmax_Euc	NA	0.84	0.92	0.64	0.87	0.85	0.82
PFS	HR	2.90	2.70	3.11	2.95	3.58	2.84	3.66
	95% CI	1.8–4.7	1.7–4.2	2.0–4.9	1.7–5.1	2.2–5.8	1.8–4.5	2.2–6.1
	*p*-value	<0.0001	<0.0001	<0.0001	0.0001	<0.0001	<0.0001	<0.0001
	Se	0.27	0.32	0.30	0.18	0.25	0.30	0.23
	Sp	0.90	0.87	0.89	0.92	0.92	0.89	0.93
OS	HR	2.66	2.60	2.90	3.11	3.10	2.85	2.93
	95% CI	1.4–4.8	1.5–4.6	1.6–5.2	1.6–6.0	1.7–5.7	1.6–5.1	1.5–5.6
	*p*-value	0.0013	0.0009	0.0003	0.0008	0.0003	0.0004	0.0011
	Se	0.28	0.33	0.31	0.20	0.26	0.31	0.22
	Sp	0.88	0.84	0.87	0.91	0.89	0.87	0.91

**Table 3:** Multivariable analysis testing of MTV, ECOG with maximal standardized distances (SDmax) calculated with each method for PFS and OS prediction. Boldface *p*-values are significant at *p* < 0.05.

Factors	Multivariate Analysis of PFS		Multivariate Analysis of OS	
	HR (95% CI)	*p*	HR (95% CI)	*p*
High SDmax_Euc	2.4 (1.5–4.0)	**0.0004**	1.8 (0.98–3.4)	0.0586
MTV > 220 cm^3^	2.1 (1.3–3.3)	**0.0027**	3.1 (1.6–6.0)	**0.0012**
ECOG 2–3	2.3 (1.4–3.8)	**0.0007**	2.3 (1.3–4.0)	**0.0062**
HighSDmax_Man	2.1 (1.3–3.4)	**0.0022**	1.7 (0.91–3.0)	0.0971
MTV > 220 cm^3^	2.0 (1.2–3.2)	**0.0052**	3.0 (1.5–6.0)	**0.0015**
ECOG 2–3	2.3 (1.4–3.7)	**0.0011**	2.2 (1.2–4.0)	**0.0076**
High SDmax_Tch	2.4 (1.5–3.9)	**0.0004**	1.9 (1.0–3.4)	**0.0433**
MTV > 220 cm^3^	2.0 (1.2–3.2)	**0.0064**	3.0 (1.5–5.8)	**0.0018**
ECOG 2–3	2.2 (1.4–3.6)	**0.0013**	2.2 (1.2–4.0)	**0.0076**
High SD_TSP	2.2 (1.2–3.9)	**0.0084**	1.9 (0.93–3.8)	0.081
MTV > 220 cm^3^	2.0 (1.2–3.2)	**0.0055**	3.0 (1.5–5.9)	**0.0018**
ECOG 2–3	2.4 (1.5–3.9)	**0.0005**	2.4 (1.3–4.2)	**0.0038**
High SDmax_Euc _Vox	2.7 (1.6–4.6)	**0.0001**	2.0 (1.0–3.7)	**0.0389**
MTV > 220 cm^3^	2.0 (1.2–3.2)	**0.0051**	3.0 (1.5–5.9)	**0.0014**
ECOG 2–3	2.3 (1.4–3.7)	**0.001**	2.2 (1.2–4.0)	**0.0073**
High SDmax_Man_Vox	2.3 (1.4–3.7)	**0.0006**	1.9 (1.1–3.5)	**0.0306**
MTV > 220 cm^3^	2.0 (1.3–3.2)	**0.0038**	3.0 (1.5–5.9)	**0.0013**
ECOG 2–3	2.3 (1.4–3.8)	**0.0006**	2.2 (1.2–4.0)	**0.0068**
High SDmax_Tch_Vox	2.8 (1.6–4.7)	**0.0002**	1.8 (0.91–3.6)	0.0911
MTV > 220 cm^3^	2.0 (1.2–3.2)	**0.0048**	3.1 (1.6–6.0)	**0.0012**
ECOG 2–3	2.2 (1.4–3.6)	**0.0012**	2.2 (1.2–4.0)	**0.0075**

## Data Availability

The data presented in this study are available on request from the corresponding author.
